# Bio‐Inspired Multiscale Design for Strong and Tough Biological Ionogels

**DOI:** 10.1002/advs.202207233

**Published:** 2023-03-11

**Authors:** Kaiyue Cao, Ying Zhu, Zihao Zheng, Wanke Cheng, Yifei Zi, Suqing Zeng, Dawei Zhao, Haipeng Yu

**Affiliations:** ^1^ Key Laboratory of Bio‐based Material Science and Technology of Ministry of Education Northeast Forestry University Harbin 150040 P. R. China; ^2^ Key Laboratory on Resources Chemicals and Materials of Ministry of Education Shenyang University of Chemical Technology Shenyang 110142 P. R. China

**Keywords:** biomimetic design, cellulose, ionogels, multiscale structure, silk fibers

## Abstract

Structure design provides an effective solution to develop advanced soft materials with desirable mechanical properties. However, creating multiscale structures in ionogels to obtain strong mechanical properties is challenging. Here, an in situ integration strategy for producing a multiscale‐structured ionogel (M‐gel) via ionothermal‐stimulated silk fiber splitting and moderate molecularization in the cellulose‐ions matrix is reported. The produced M‐gel shows a multiscale structural superiority comprised of microfibers, nanofibrils, and supramolecular networks. When this strategy is used to construct a hexactinellid inspired M‐gel, the resultant biomimetic M‐gel shows excellent mechanical properties including elastic modulus of 31.5 MPa, fracture strength of 6.52 MPa, toughness reaching 1540 kJ m^−3^, and instantaneous impact resistance of 3.07 kJ m^−1^, which are comparable to those of most previously reported polymeric gels and even hardwood. This strategy is generalizable to other biopolymers, offering a promising in situ design method for biological ionogels that can be expanded to more demanding load‐bearing materials requiring greater impact resistance.

## Graphical abstract

A high‐performance biomimetic ionogel is developed by in situ multiscale design of silk fiber in a cellulose‐ions matrix. The biomimetic ionogel shows high ionic conductivity of 49.6 mS cm^−1^ and ultra‐strong mechanical properties with a fracture strength of 6.5 MPa and impact resistance as high as 3.07 kJ m^−1^, holding great application potential in flexible bioelectronics and smart protective devices.

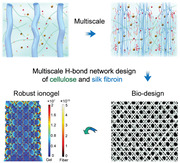

## 1 Introduction

Developing high‐performance ionogels has become one of the most exciting frontiers in materials science field. Their bioaffinity, flexibility, and ionic conductivity give ionogels promising applications in flexible sensors, intelligent electronics, e‐skins, wearable monitoring devices, and soft robots.^[^ ^1^, ^2^, ^3^ ^]^ However, most ionogels show weak mechanical properties, such as fracture strength of <1 MPa, modulus of <5 MPa, and toughness of ≈500 kJ m^−3^.^[^ ^4^, ^5^, ^6^ ^]^ These have literally limited their expansion in application fields. To date, a variety of structure‐designing methods such as multi‐component polymerization, the construction of sacrificial bonds, and the addition of physical fillers have been investigated; however, these measures have several limitations, including poor interface compatibility, a trade‐off between flexibility and conductivity, or the premature failure of strength and endurance.^[^ ^7^, ^8^, ^9^, ^10^ ^]^ Therefore, the strategy of developing an ionogel with good conductivity, flexibility, and simultaneous with mechanical robustness is challenging.

Hexactinellid sponge (*Euplectella aspergillum*), living in the abyssal sea of ≈8500 m, has attracted attentions due to its unique body skeleton, which is composed of nanoscale silicon filaments and microscale hydrated silicon fibers. These form a paired‐parallel and diagonal grid architecture across multiple length scales, endowing the hexactinellid sponge with exceptional mechanical robustness and resistance to seawater up to 85 atmospheres.^[^ ^11^, ^12^, ^13^ ^]^ Inspired by this skeleton, a sponge‐biomimetic square lattice material was 3D‐printed by Matheus and co‐workers, which showed a high buckling resistance to uniaxial impact.^[^ ^14^ ^]^ Therefore, integrating such a multiscale‐derived bio‐based structure into an ionogel is expected to improve the mechanical properties and robustness. The key to accomplishing this lies in the search for two heterogeneous yet structurally complementary materials: one must serve as the continuous phase to provide a stable, flexible, and scalable matrix for structural support; the other must undergo a nanofibrillation transformation to develop a multilevel structure to serve as an optimal reinforcement.

In our earlier work, cellulose as a biomass resource was dissolved in an ionic liquid and transformed into flexible 2D ionogel materials with abundant anions/cations and high conductivity.^[^ ^15^ ^]^ It exhibited promising applications in biosensors, e‐skins, flexible electronics, and soft robots.^[^ ^16^, ^17^, ^18^, ^19^, ^20^ ^]^ Silk fiber is another bioactive renewable material with an intrinsically and exquisitely hierarchical structure composed of microfibers, nanofibrils, and molecular‐scale protein/polypeptide chains.^[^ ^21^, ^22^, ^23^, ^24^ ^]^ The rational integration of these two biomaterials both from the component aspect and from the structure and morphology aspect will make their derived ionogels more powerful and biocompatible. However, such high‐performance biological ionogels generated from cellulose and silk fibroin have not been developed.

**Figure 1:**
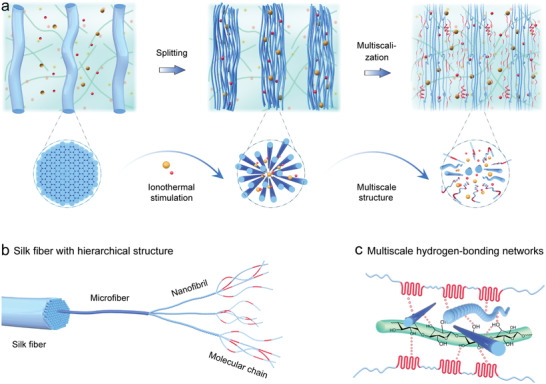
Creating the M‐gel by inducing silk fibers into Cel‐gel matrix. a) Schematic illustration of the nanofibrillation and molecularization of silk fibers in Cel‐gel. b) Intrinsically hierarchical structures of a silk fiber. c) Multiscale H‐bonding topological network benefits the physicochemical enhancement of Cel‐gel.

Here, we developed an ionogel with a bio‐inspired multiscale structure by guiding the in situ splitting (nanofibrillation) and molecularization of silk fibers in a cellulose‐ions matrix. We conceived a cellulose‐ions gel (Cel‐gel) by dissolving cellulose in 1‐butyl‐3‐methylimidazolium chloride ([Bmim]Cl), which provides a supramolecular matrix phase to incorporate the silk fibers (**Figure** **1**a). Along with the chemical crosslinks within silk fibers are gradually broken by the surrounding [Bmim]^+^/Cl^−^ ions, each silk fiber in Cel‐gel will split into microfibers, then more nanofibrils, and even fibroin chains along with the thermal reaction time. These silk fibers‐derived microfibers, nanofibrils, and fibroin chains constitute a multiscale hydrogen‐bonding (H‐bonding) system that can act as physicochemical phase for the Cel‐gel reinforcement (Figure 1b,c). Based on this design strategy, a multiscale‐structured ionogel (M‐gel) with biomimetic architecture was developed to achieve the optimal structural enhancement objective. The resulting M‐gel showed its ultra‐strong mechanical properties such as tensile strength of over 6.5 MPa, elastic modulus of reaching 31.5 MPa, and with impact resistance of 3.07 kJ m^−1^, and high ionic conductivity of 49.6 mS cm^−1^, which are crucial factors in advanced applications of flexible and robust electronics.

## 2 Results and Discussion

### 2.1 Multiscale Structure Construction

**Figure 2:**
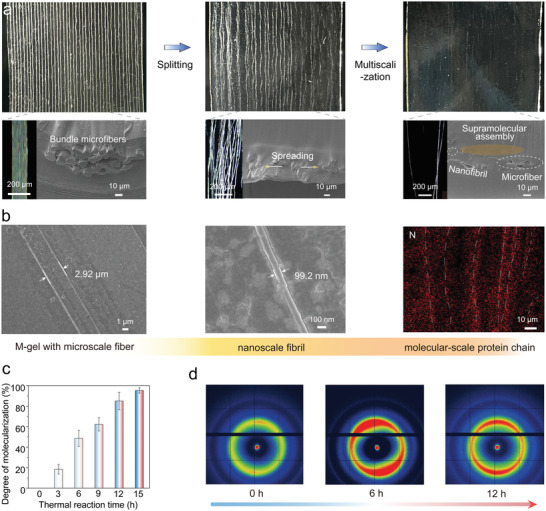
In situ integration of silk fibers into Cel‐gel to form M‐gel. a) Optical microscopy and b) SEM images showing the transformation of silk fibers during the thermal reaction process. c) Degree of molecularization of silk fibers in M‐gel. d) In situ SAXS patterns of the M‐gel at different thermal reaction time.

First, the Cel‐gel with active hydroxyl groups and ions served as a matrix and provided abundant [Bmim]^+^ and Cl^−^ for the integration of silk fibers (Figure S1, Supporting Information). Generally, a single silk fiber contains dozens of microfibers (Figure S2, Supporting Information) and hundreds of nanofibrils. When the silk fibers were treated by the surrounding [Bmim]^+^ and Cl^−^ ions at 80 °C, each pristine silk fiber was split into finer microfibers (Figures S3 and S4a, Supporting Information), which simultaneously spread out of the fiber (**Figure** **2**a). Upon prolonging the thermal reaction time, the ends of these microfibers were further split into more nanofibrils and even fibroin chains (Figure 2b). The resultant nanofibrils and fibroin chains were likely to crosslink/assemble with cellulose chains via H‐bond to construct supramolecular topological networks.

Through experimental verification and small‐angle X‐ray scattering (SAXS) analysis, the scattering peak intensity of M‐gel gradually increased when the reaction time was less than 6 h (Figure S4b, Supporting Information), indicating that silk fibers were split into microfibers and nanofibrils. Through calculating the change in transmittance value (*T*%) of M‐gel over different thermal reaction time (Figure S4c, Supporting Information), we could estimate the degree of molecularization of silk fibers in M‐gel between 0 and 100% (Figure 2c). For example, when thermal reaction time of 6 h, the silk fibers in M‐gel showed the degree of molecularization of 48.6%, which indicated that nearly half of silk fibers were converted into nanofibrils and fibroin chains (Figure 2c).

As shown in Figure 2d, the SAXS patterns of M‐gels became brighter and stronger during thermal reaction time of 6 h. It was due to the strengthening H‐bond networks between fibroin chains, silk fibroins, and cellulose molecular chains, leading to the increase of electron density difference in the multiscale interfaces of M‐gel. Upon further prolonging the reaction time, the silk fibers would be completely dissolved in the M‐gel with degree of molecularization of ≈100% (Figure S4a, Supporting Information), resulting in a homogeneous supramolecular system and showing relatively weaker scattering intensity in the SAXS pattern (Figure S5, Supporting Information). Through above analysis, the optimal thermal reaction time for developing M‐gel was determined to be 6 h, and the transformed multiscale structure in this state could greatly improve the mechanical properties of M‐gel.

### 2.2 Multiscale Structure Evolution Analysis

**Figure 3:**
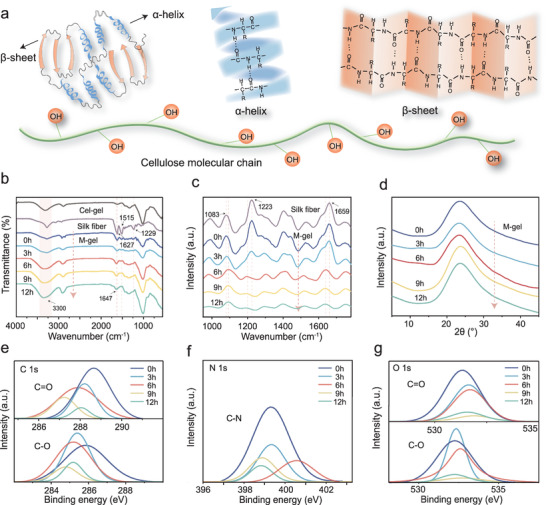
Spectroscopic analyses of the evolution of M‐gel. a) Molecular structure of the silk fibroin and cellulose. b–d) FTIR, Raman, XRD spectra, and high‐resolution XPS elemental spectra of e) C 1s, f) N 1s, and g) O 1s of samples, respectively.

A series of spectroscopic analyses including Fourier transform infrared spectroscopy (FTIR), Raman spectroscopy, X‐ray diffraction (XRD), and X‐ray photoelectron spectroscopy (XPS) were performed to investigate changes in the structure of M‐gel. Cellulose had a degree of polymerization of 1500 and showed long molecular chains rich with hydroxyl groups (—OH). Silk fibroin was comprised of polypeptide molecules with amino and carboxyl groups, displaying random coils, *α*‐helical, and *β*‐folded conformations (**Figure** **3**a). As shown in Figure 3b, the absorption peaks at 1627, 1515, and 1229 cm^−1^ belonging to the *β*‐fold conformation of silk fibroin^[^ ^25^, ^26^ ^]^ became weaker upon increasing the thermal reaction time. The Raman spectrum showed *β*‐fold characteristic peaks at 1083, 1223, and 1659 cm^−1^, which also showed weakened intensities (Figure 3c). These indicated the gradual dissociation of the *β*‐fold structure of silk fibroin and its transformation into micro‐/nanofibers and protein molecules. These changes were attributed to the thermal swelling effect and breakage of the internal H‐bonds of silk fibroin induced by [Bmim]^+^/Cl^−^ ions reaction. The stronger and sharper change in the FTIR stretching vibration peak of ‐OH at 3300 cm^−1^ demonstrate that the molecularized fibroin chains formed denser supramolecular H‐bonds with cellulose molecules (Figure 3b).

In the XRD patterns in Figure 3d, no significant changes were found on the crystal form during the whole process, but the diffraction peak intensity increased as the thermal reaction proceeded. This was due to the splitting of silk fiber into microfibers and nanofibrils, leading to more microcrystals exposed and partial amorphous area dissolved by ions. In the XPS spectra (Figure 3e–g), the peaks of C=O/C—O (C 1s, Figure 3e) shifted to a lower binding energy during the reaction, indicating that partial splitted nanofibrils were molecularized to fibroin chains. In the N 1s and O 1s spectra (Figure 3f,g), the binding energy of the peaks of C−N (N1s) and C=O/C−O (O 1s) gradually increased over the 6 h of thermal reaction. This demonstrated that the multiscale H‐bond networks between microfibers, nanofibrils, protein molecules, and cellulose molecules became increasingly dense and strong. These changes are expected to give the M‐gel strong mechanical properties. However, an excessive reaction time was detrimental to the formation of multiscale structures and damaged the H‐bond topological networks, as evidenced by the gradual decrease in the binding energy of C—N (N 1s) when stimulated for 9 and 12 h. This degraded the mechanical properties of the M‐gel. As shown in Figure S6, Supporting Information, the tensile strength of M‐gel was only 0.82 MPa when it was subjected to stimulation for 15 h.

### 2.3 Mechanical Properties of M‐Gels

**Figure 4:**
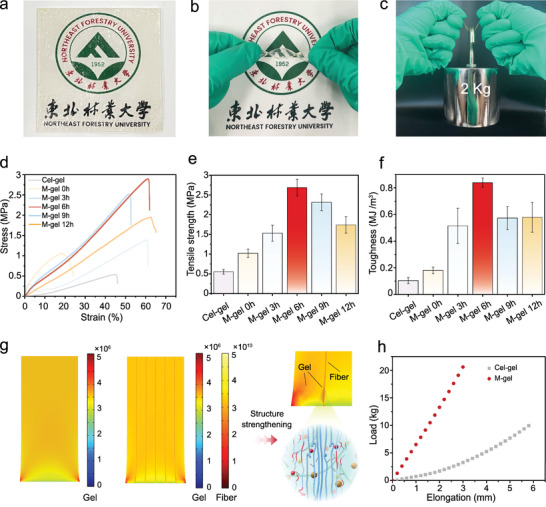
Investigation on the mechanical properties of M‐gel. Optical photographs showing the a) transparency, b) flexibility, and c) robustness of the M‐gel. d) Tensile stress–strain curves of M‐gels obtained from different thermal reaction time. Comparison of e) tensile strength and f) toughness of the M‐gels obtained from different thermal reaction time. g) Stress distribution nephogram of Cel‐gel and M‐gel obtained by finite element simulations. h) Elongation–load curves of Cel‐gel and M‐gel obtained by finite element simulations. The Northeast Forestry University logo is reproduced with permission.

Compared with other structural reinforcement methods, this multiscale structural design strategy greatly improved the mechanical properties of the M‐gel. As shown in **Figure** **4**a–c, the M‐gel with a high transparency and flexibility could easily load a metal weight of up to 2 kg, which is 8333 times its own weight. This was a far greater value than that of the Cel‐gel without a multiscale structure (Figure S7, Supporting Information). The mechanical properties of the M‐gel could also be tuned by adjusting the thermal reaction time (Figure 4d). The M‐gel with degree of molecularization of ≈48.6%, obtained from a 6 h thermal reaction time, showed a high tensile strength of 2.69 MPa, a Young's modulus of 7.3 MPa, and a toughness of 850 kJ m^−3^, respectively (Figure 4d–f). These values were all significantly superior to those (tensile strength of 0.55 MPa, Young's modulus of 0.75 MPa, and toughness of 120 kJ m^−3^) of the Cel‐gel (Figure S8, Supporting Information). By observing the tensile fracture section of M‐gel (Figure S9, Supporting Information), we found that a sufficient thermal reaction time such as 6 h greatly improved the interfacial compatibility and stability of multiscale silk fibers and the cellulose‐silk protein supramolecular system, thus strengthening the properties of the resulting ionogel. Both the silk fibers in completely molecularization state (degree of molecularization of ≈100%) and just filling state (non‐molecularization) had failed to improve the mechanical performances of M‐gel, with the mechanical tensile strength of only 0.85 MPa (Figure 4e) and 1.02 MPa (Figure S6, Supporting Information), respectively.

Finite element simulations of the stress distribution nephogram (Figure 4g and Figure S10, Supporting Information) showed that the M‐gel presented a better stress‐bearing and dispersing capacity compared with the original Cel‐gel due to its unique multiscale structure strengthening. Microscale fibers and their derived nanofibrils in the M‐gel played structural skeleton roles and strengthened the flexible supramolecular matrix. Supramolecular topological networks between cellulose molecules and silk protein molecules played the chemical crosslinking roles and enhanced the interfacial compatibility and robustness between the multiscale structures. The M‐gel showed a load‐bearing capability more than 6 times greater than that of the original Cel‐gel at elongation of 3 mm (Figure 4h).

### 2.4 Biomimetic Design of M‐Gels

**Figure 5:**
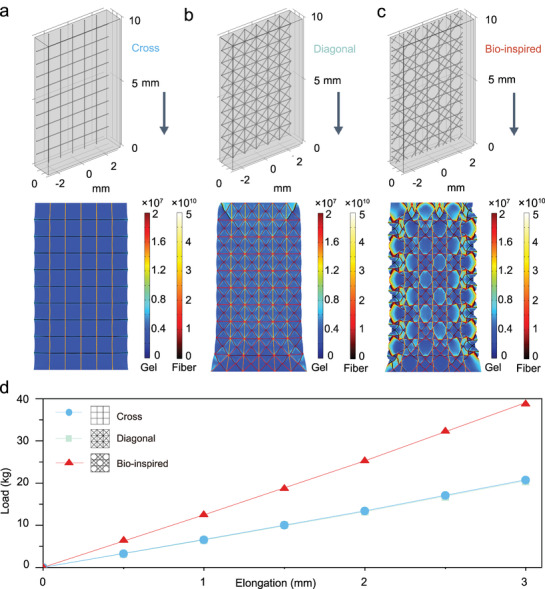
Finite element simulations on tensile properties of the M‐gels with different grid structures. a–c) Model design and stress distribution nephogram of the M‐gels with cross, diagonal, and hexactinellid bio‐inspired grid structures, respectively. d) Finite element simulated elongation‐load curves of the bio‐inspired M‐gels with different grid structures.

The integration of Cel‐gel and silk fibers provides a good foundation for preparing the multiscale structural ionogel; however, further optimization using a biomimetic design can further improve the mechanical properties and robustness of the M‐gel. The evolutionary process of the hexactinellid sponge has given it a perfect tissue architecture of a paired‐parallel and cross‐diagonal grid, accompanied by excellent mechanical properties that allow it to withstand external impacts.^[^ ^12^ ^]^ Through finite element simulations (**Figure** **5**a–c), we confirmed that the grid structure of hexactinellid sponge greatly improved the mechanical properties of M‐gel, especially the impact resistance and toughness, compared with the cross‐ and diagonal‐grid structure (Figure S11, Supporting Information). Figure 5d shows that this biomimetic structure gave the M‐gel a theoretical high load‐bearing of more than 40 kg, which is twice as much as the other two grid modes.

**Figure 6:**
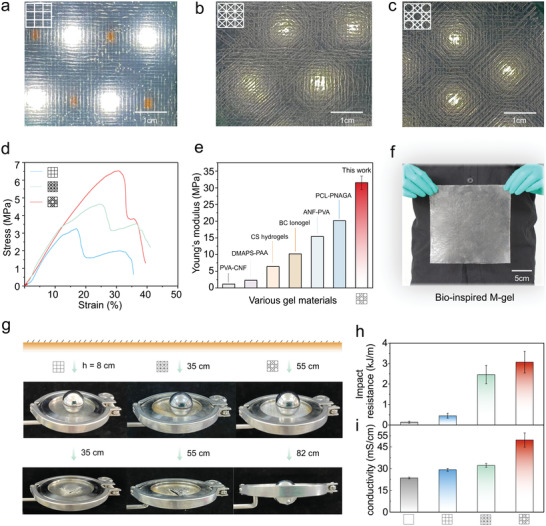
Mechanical properties and applications of the biomimetic M‐gels. a–c) Optical images of M‐gels with cross, diagonal, and hexactinellid biomimetic grid structures, respectively. d) Tensile stress–strain curves of the M‐gels with different structures. e) Comparison of the Young's modulus of the biomimetic M‐gel with other reported gel materials. f) Optical image of the hexactinellid biomimetic M‐gel with a scalable size. g) Free‐fall impact resistance of the M‐gels with different grid structures. The weight of the iron ball was 256 g. h) Comparison of the impact resistance between the biomimetic M‐gel and other kinds of M‐gels. i) Biomimetic M‐gel showing the higher ionic conductivity.

Three modes of M‐gels with cross‐, diagonal‐, and biomimetic skeletal structures were fabricated as shown in **Figure** **6**a–c. Tensile mechanical property tests (Figure 6d) showed that the hexactinellid bio‐inspired M‐gel exhibited optimal mechanical properties with a tensile strength of 6.52 MPa and a toughness of over 1500 kJ m^−3^ (Figure S12, Supporting Information), which were superior to those of cross‐ and diagonal‐structured M‐gels. This biomimetic M‐gel showed remarkable stress buffering and damage resistance characteristics (Figure 6d) compared with those of single parallel, cross, and diagonal patterns. As shown in Figure 6e, the biomimetic M‐gel had a high Young's modulus of 31.5 MPa, which was better than that of most previously reported gel materials.^[^ ^9^, ^27^, ^28^, ^29^, ^30^, ^31^, ^32^ ^]^ In addition, we could freely enlarge the size of this biomimetic M‐gel according to the requirements of actual applications (Figure 6f).

From the free‐fall test with an iron ball (weight of 256 g), our biomimetic M‐gel exhibited strong mechanical impact resistance (Figure 6g), with the highest impact resistance of 3.07 kJ m^−1^ (Figure 6h), which was superior to that of other M‐gels and even better than that of hardwood board (2.63 kJ m^−1^). Meanwhile, this biomimetic high‐strength M‐gels also presented excellent ionic conductivity of up to 49.6 mS cm^−1^ (Figure S13, Supporting Information), which was superior to that of most conductive gel materials (Figure 6i).^[^ ^33^, ^34^, ^35^, ^36^ ^]^ Compared to pure [Bmim]Cl^[^ ^37^, ^38^ ^]^ with ionic conductivity of 0.0714 mS cm^−1^ (Figure S14, Supporting Information), the M‐gel showed a nearly 700 times higher ion conductivity than it. This was due to its unique multiscale H‐bondiqg networks for M‐gel, which effectively improved the mobility of [Bmim]^+^/Cl^−^ ions via H‐bond guidance.^[^ ^15^ ^]^ Benefiting from this biomimetic multiscale structural design, we can greatly enhance the electrical or mechanical properties of gel materials without increasing temperature or/and ions concentration, showing the competitively comprehensive advantages (Table S1, Supporting Information). As a proof‐of‐concept demonstration, the M‐gel with high ionic conductivity and robust mechanical properties had the advanced applications in an intelligent device and a smart bioelectronic sensor for various stimulus signals (Figure S15, Supporting Information).

## 3 Conclusion

Here, we provided a bio‐inspired structure enhancement strategy for developing high‐strength biological ionogels by integrating green and biocompatible silk fibroin and cellulose. Both the hierarchical structure of silk fibroin and the unique thermal effect of the cellulose‐ions matrix endowed the ionogel materials with multiscale structures. Through the bio‐inspired reinforcement design and the construction of cross‐scale physicochemical topological networks, the resultant M‐gel presented excellent mechanical properties, including a high tensile strength of 6.52 MPa, a robust Young's modulus of 31.5 MPa, and an excellent toughness of over 1500 kJ m^−3^, an outstanding impact resistance of over 3 kJ m^−1^, and a high ionic conductivity of 49.6 mS cm^−1^, 700 times higher ion conductivity than pure [Bmim]Cl and surpassing most reported polymeric ionogels. This work will open a new perspective for biomimetic structure design to develop functional and innovative ionogel materials with superior mechanical properties. This will help expand their potential applications in the fields of intelligent protective devices and robust bioelectronics.

## 4 Experimental Section

### Materials and Chemicals

Cellulose was extracted from poplar wood flour with a mean degree of polymerization of 1500, a crystallinity of 64%, and a diameter within 50–250 µm.^[^ ^39^ ^]^ 1‐Methylimidazole (99%) and 1‐chlorobutane (99.8%) were obtained from Aladdin (Shanghai, China). The silk fibers (water content of 5.5 wt%) were purchased from Simeite Biotechnology Co., Ltd. (Suzhou, China). The 1‐butyl‐3‐methylimidazolium chloride ([Bmim]Cl) was obtained using a previously reported method.^[^ ^37^ ^]^

### Preparation of Cel‐Gel and M‐Gel

Before constructing the samples, the silk fibers, cellulose, and [Bmim]Cl were treated in a drying oven at 80 °C for 48 h to remove water. A viscous homogeneous system of cellulose/[Bmim]Cl (cellulose of 5 wt%) was prepared by thermal dissolution at 80 °C for 6 h. The cellulose/[Bmim]Cl system was spread onto a smooth glass plate and placed in a vacuum oven (0.01 MPa) at 85 °C for 24 h to remove bubbles. Then, the cellulose‐ion gel matrix (Cel‐gel) was obtained when placing the de‐bubbled cellulose/[Bmim]Cl system at ambient conditions (temperature of 25–30 °C and relative humidity of 45–55%) for 20 min. The silk fibers were directly embedded in the Cel‐gel with designable patterns (the spacing between each embedded silk fiber was 1 mm) and treated in a blast‐drying oven at 80 °C for different times. Finally, the produced ionogel was moved to ambient conditions (temperature of 25–30 °C and relative humidity of 45–55%) for 1 h to absorb water molecules for the gelation to obtain the M‐gel (water content ≈14 wt%). The amounts of these components and processing conditions were listed in Table S2, Supporting Information.

### Calculate the Degree of Molecularization of Silk Fibers in M‐Gel

The light transmittance value (*T*%) of M‐gel at different thermal reaction time was tested by the JDY‐100 digital hazemeter (Jindouyun Precision Instrument Co., LTD, Jiangsu, China) at wavelength of 550 nm. The degree of molecularization of silk fibers is calculated by the following equation:

(1) $$\mathrm{DM}=\left(T_{M-\mathrm{gel}-X}-T_{\left(\mathrm{silk}\;\mathrm{fiber}\right)}\right)\times 100\%/\left(T_{\left(M-\mathrm{gel}-20\right)}-T_{\left(\mathrm{silk}\;\mathrm{fiber}\right)}\right)$$

where *T*_(M‐gel−_ *_X_*_)_ is the *T*% of M‐gel treated at thermal reaction time of *X* h, *T*_(silk fiber)_ is the *T*% of original M‐gel with silk fibers, and *T*_(M‐gel−20)_ is the *T*% of the M‐gel by complete molecularization (thermal reaction time for 20 h).

### Finite Element Simulations

The finite element simulations were carried out with COMSOL Multiphysics software (version 6.0.0.318). The 3D steady‐state simulation was based on the internal structural mechanics module of the gel. The core physical relationships were based on Hooke's law, and the nonlinear mechanics were based on Green–Lagrange finite strain theory.

### Impact Resistance Test

Impact resistance tests on M‐gels with cross, diagonal, and biomimetic grid structures using a homemade setup on which a round clamping device was used to fix the M‐gels. The kinetic energy loss after the iron ball struck through the gel was used to determine the impact energy absorption of the M‐gel. The energy absorption equation was *m*(*V*_1_^2^−*V*_2_^2^)/2*t*, where *m* is the mass of the iron ball; *t* is the thickness of the M‐gel; *V*_1_ and *V*_2_ are the velocities of the iron ball before and after striking through the M‐gel.

### Ionic Conductivity Measurement

First, two nickel sheets (with width of 1 cm) served as current collectors were sandwiched on both sides of the sample. Two copper wires were attached to both ends of current collectors and then connected to a CHI760e electrochemical workstation (Chenhua Instruments, Shanghai, China), measuring the AC impedance spectrum (AC amplitude of 1 mV, frequencies between 100 mHz and 1 MHz). The bulk resistance of samples could be measured by EIS. The ionic conductivity of sample was calculated using the equation of *σ* = *L*/(*R*_b_ × *A*), where *L* (cm) denotes the thickness of the sample, *A* (cm^2^) denotes effective cross‐sectional area of the sample, and *R*_b_ (Ω) denotes bulk resistance of the sample.

### Sensing Signals Monitoring Test

The biomimetic M‐gel was connected to a CHI760e electrochemical workstation (Chenhua Instruments, Shanghai, China). The current waveforms of M‐gel sensing included clicking, breathing, and bending were assessed by measuring the amperometric *I*–*t* curve parameters with an initial potential of 0 V at room temperature.

## Conflict of Interest

The authors declare no conflict of interest.

## Author Contributions

K.C. and Y.Z. contributed equally to this work. H.Y. and D.Z. supervised the project. H.Y. and D.Z. designed the experiments. K.C. and Y.Z. carried out most of the experiments. Z.Z. and W.C. contributed to the results discussion. Y.Z., W.C., Y.Z., and S.Z. contributed to part of the experiment. K.C., D.Z., and H.Y. collectively co‐wrote the paper. All authors commented on the final manuscript.

## Supporting information

Supporting Information

## Data Availability

The data that support the findings of this study are available in the supplementary material of this article.;
