# Supplementary material for: Bio‐Inspired Multiscale Design for Strong and Tough Biological Ionogels
Source: Adv Sci (Weinh). 2023 Mar 11;10(13):2207233. doi: 10.1002/advs.202207233 (PMC10161113; doi:10.1002/advs.202207233)
Supplement: Supplementary file 1 — Supporting Information [file ADVS-10-2207233-s001.pdf]

Supporting information

Bio-inspired Multiscale Design for Strong and Tough Biological Ionogels

Kaiyue Cao [1,#], Ying Zhu[1,#], Zihao Zheng[1], Wanke Cheng[1], Yifei Zi[1], Suqing Zeng[1], Dawei Zhao[1,2]*, Haipeng Yu[1]*

1. Key Laboratory of Bio-based Material Science and Technology of Ministry of Education, Northeast Forestry University, Harbin 150040, P. R. China
2. Key Laboratory on Resources Chemicals and Materials of Ministry of Education, Shenyang University of Chemical Technology, Shenyang 110142, P. R. China

I. Supplementary Methods

Characterization. The microstructure of sample was characterized by the JSM-7500F scanning electron microscope (SEM, Hitachi, Tokyo, Japan) at an operating voltage of 10 kV. Optical photographs and micrographs of samples were taken through polarized light microscopy (Mingmei Optoelectronic Technology Co., Ltd., Guangzhou, China) with the M-Shot Image Analysis System V1.1. Fourier-transform infrared (FTIR) spectra of samples were obtained by a Nicolet 6700 FTIR instrument (Thermo Fisher Scientific Inc., Waltham, MA, USA). All spectra were measured in ATR mode, with data recorded in the range of 600–4000 cm^{-1} over 32 scans with a resolution of 4 cm^{-1}. X-ray photoelectron (XPS) spectra of samples were obtained by the ESCALAB 250 XI (Thermo Scientific, USA) equipped with monochromator Al target (λ = 0.05 eV). Raman spectra were performed using a Renishaw inVia Raman microscope (Renishaw, inVia, UK) equipped with a 785 nm laser, a water immersion objective (Leica, 63×, NA = 0.9), and a 1200 l/mm grating. X-ray diffraction (XRD) patterns of samples were measured by a D/max 2200 X-ray diffractometer (Rigaku, Tokyo, Japan) equipped with Ni-filtered Cu-Kα radiation (λ = 0.154 nm). The samples were scanned within 2θ of 5–90° at 40 kV and 30 mA at a scanning rate of 2° min^{-1}. The small angle X-ray scattering (SAXS) of samples was measured using the SAXSess mc2 instrument with Cu Kα X-ray radiation and a wavelength of 0.154 nm (Anton Paar, Graz, Austria). The sample-to-detector distance was 2658.5 mm and exposure time was 5 min. All mechanical properties of the M-gel were measured using an Instron 5569 universal testing machine (Instron Corp., Canton, MA, USA) at a tensile speed of 5 mm. min^{-1}.

II. Supplementary Figures

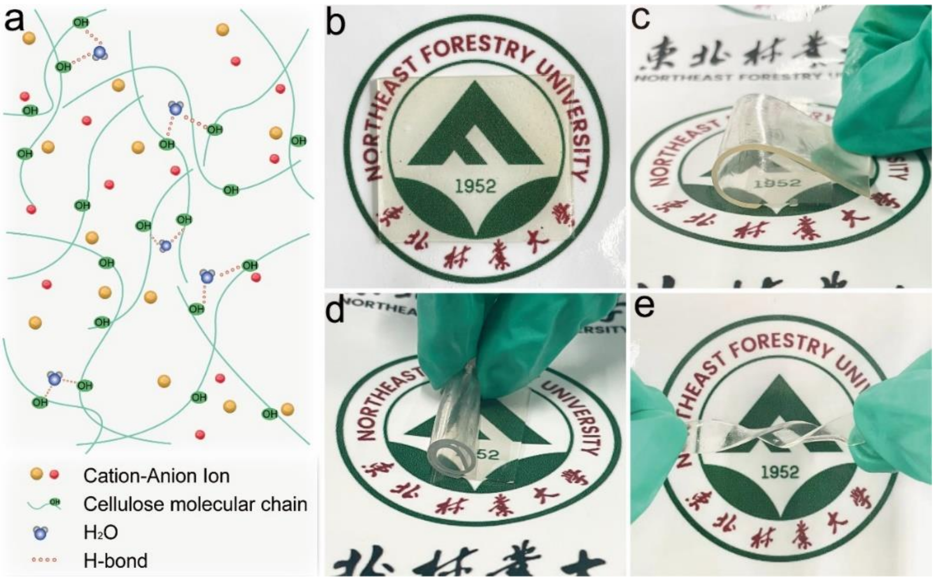

Figure S1. Cel-gel serves as a supramolecular matrix for the incorporation of silk fibers. (a) Dynamic H-bonding topological networks of the Cel-gel. (b–e) Optical photographs of the Cel-gel showing good transparency and flexibility.

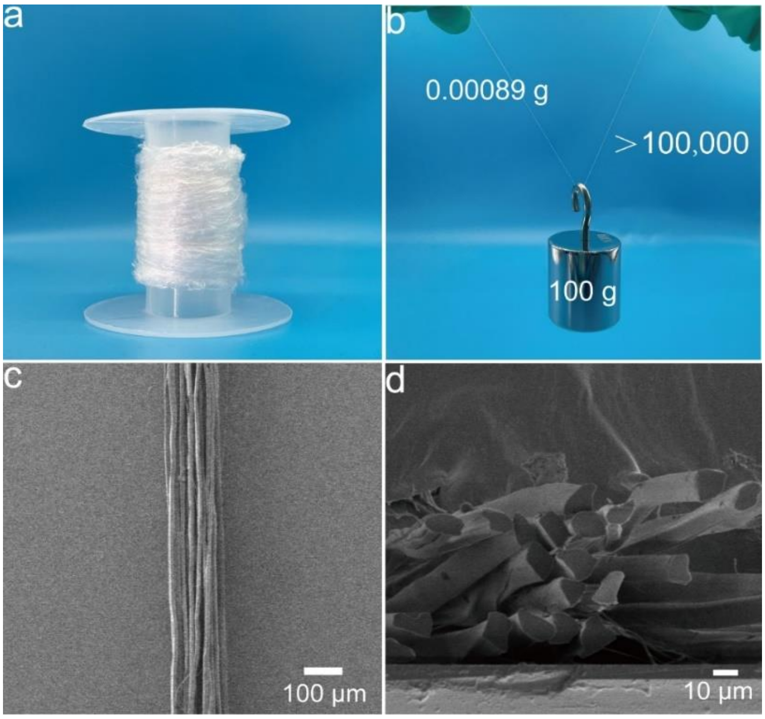

Figure S2. Silk fiber with strong mechanical properties and containing dozens of microfibers. (a) Optical picture of a silk fiber. (b) Optical picture showing a single silk fiber for lifting 100 g weight (>100,000 times of its self-weight). (c,d) Surface and cross-sectional SEM images of the microfibers from a single silk fiber during thermal reaction process.

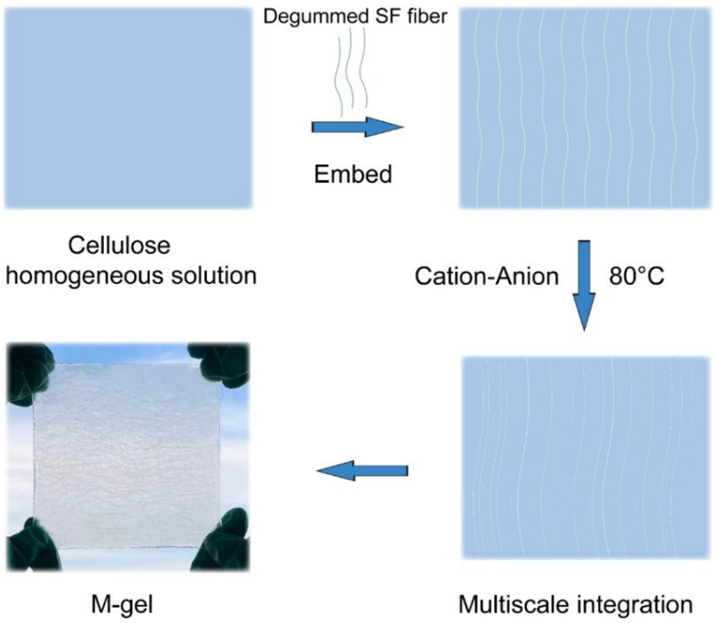

Figure S3. Preparation and construction process of the M-gel.

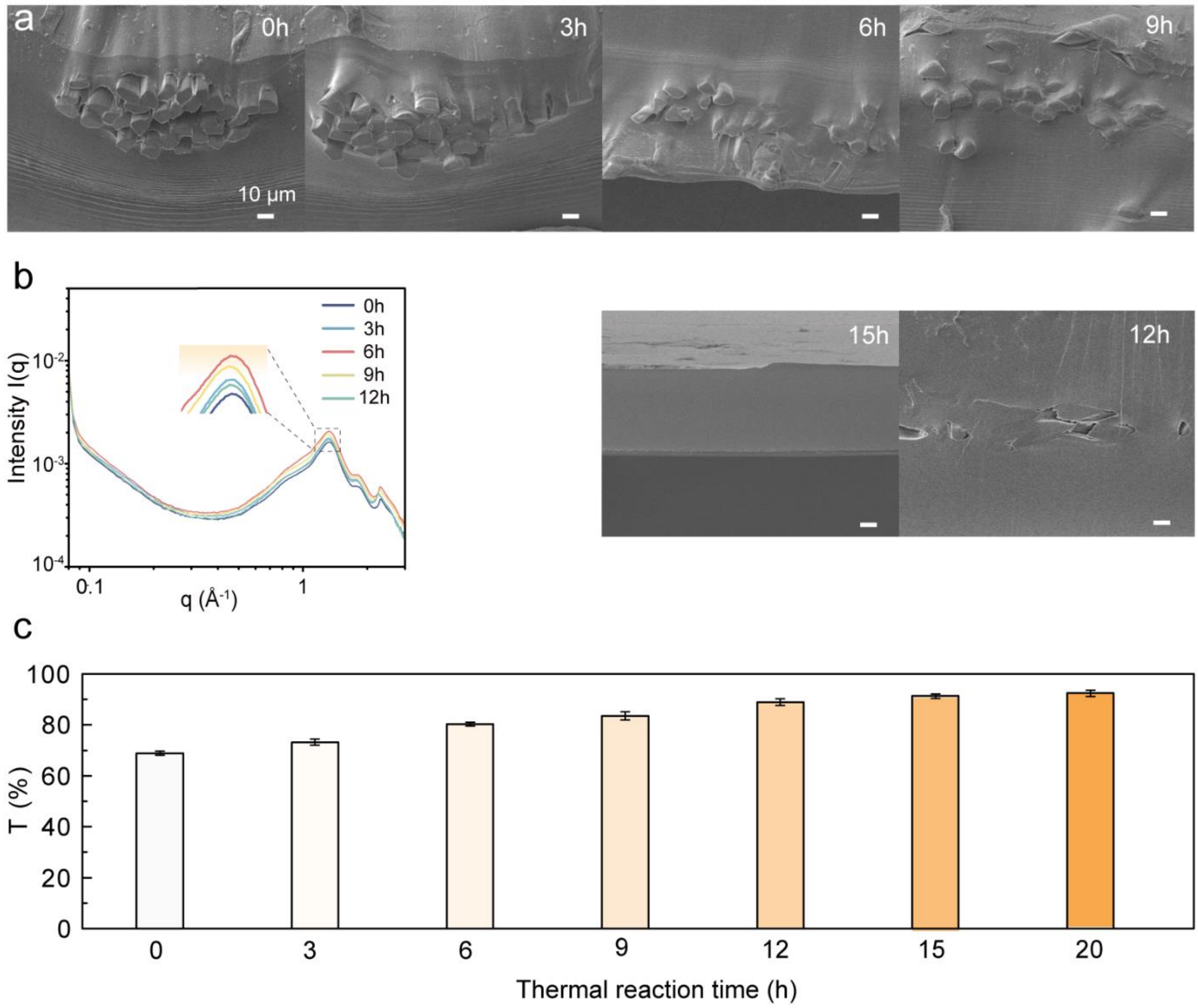

Figure S4. (a) SEM images of the silk fiber embedded in M-gel corresponding to different thermal reaction time. (b) *In situ* SAXS curves of the M-gel at different thermal reaction time. (c) Light transmittance (T %) of the M-gel during its different thermal reaction time.

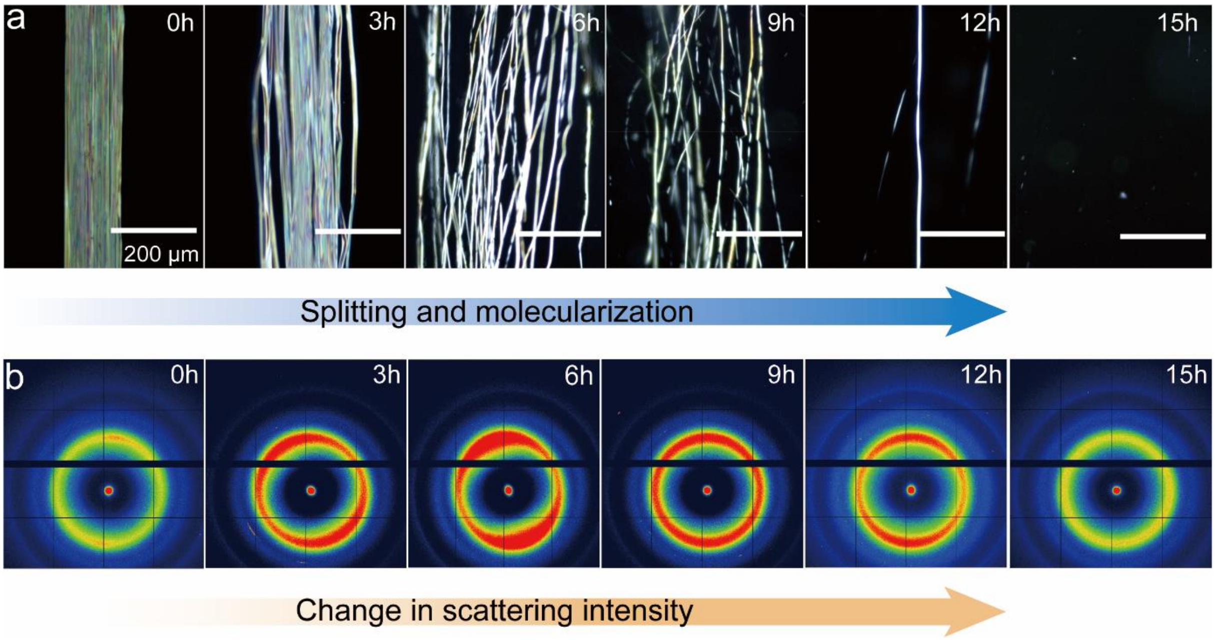

Figure S5. (a) Real-time optical microscopy photographs of a silk fiber with the thermal reaction time. (b) Real-time 2D SAXS patterns of the M-gel during different thermal reaction periods.

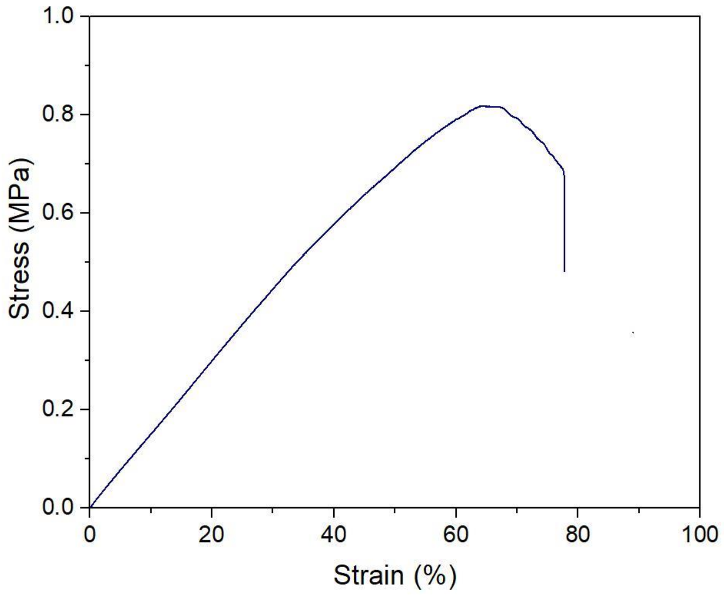

Figure S6. Tensile stress-strain curve of the M-gel resulting from thermal reaction of 15 h.

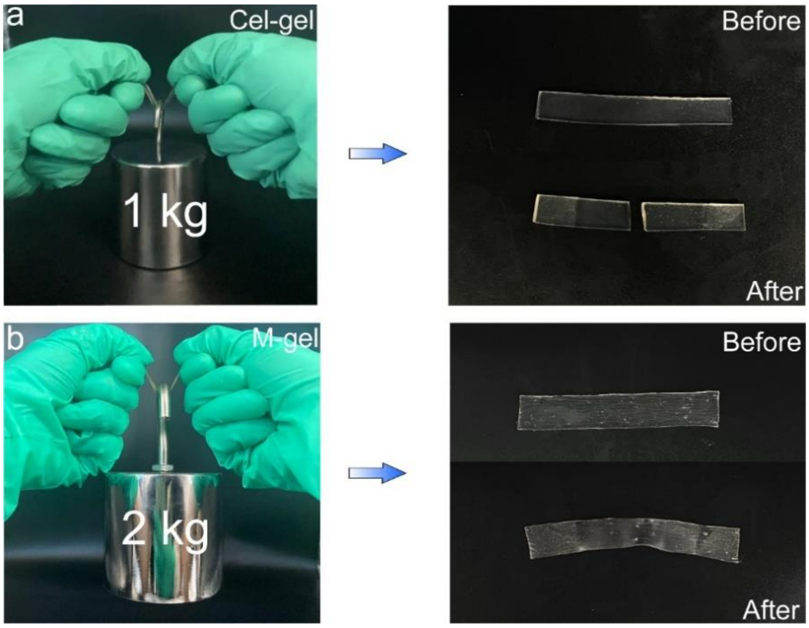

Figure S7. Comparison of the mechanical behaviors between Cel-gel and M-gel. (a) Optical photographs of the Cel-gel used to lift a weight of 1 kg. (b) Optical photographs of the M-gel lifting a weight of 2 kg successfully.

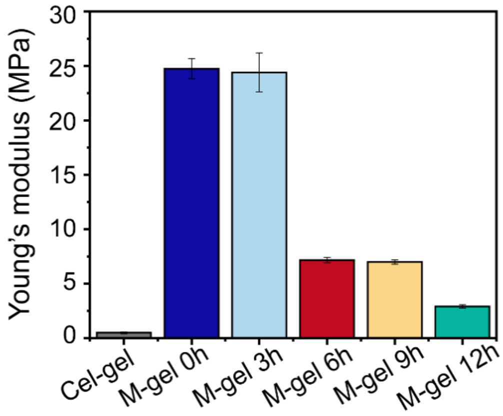

Figure S8. Comparison of the Young's modulus of the M-gels from different thermal reaction time.

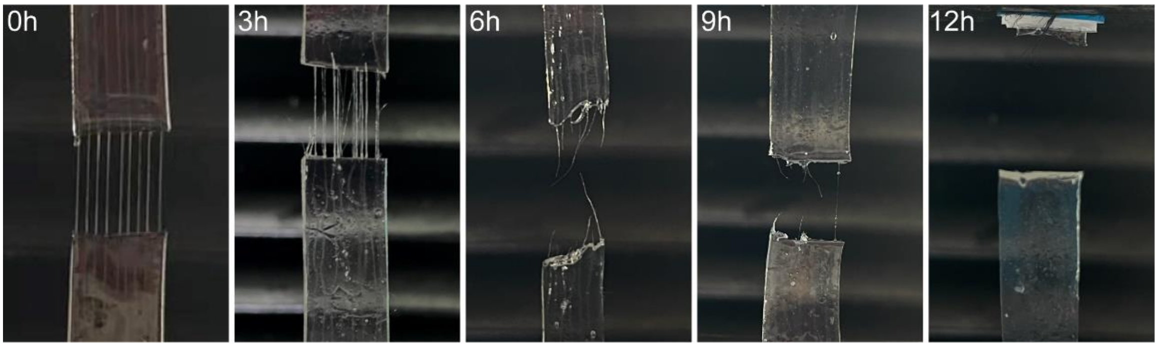

Figure S9. Comparison of fracture morphology of the M-gels treated with different thermal reaction time.

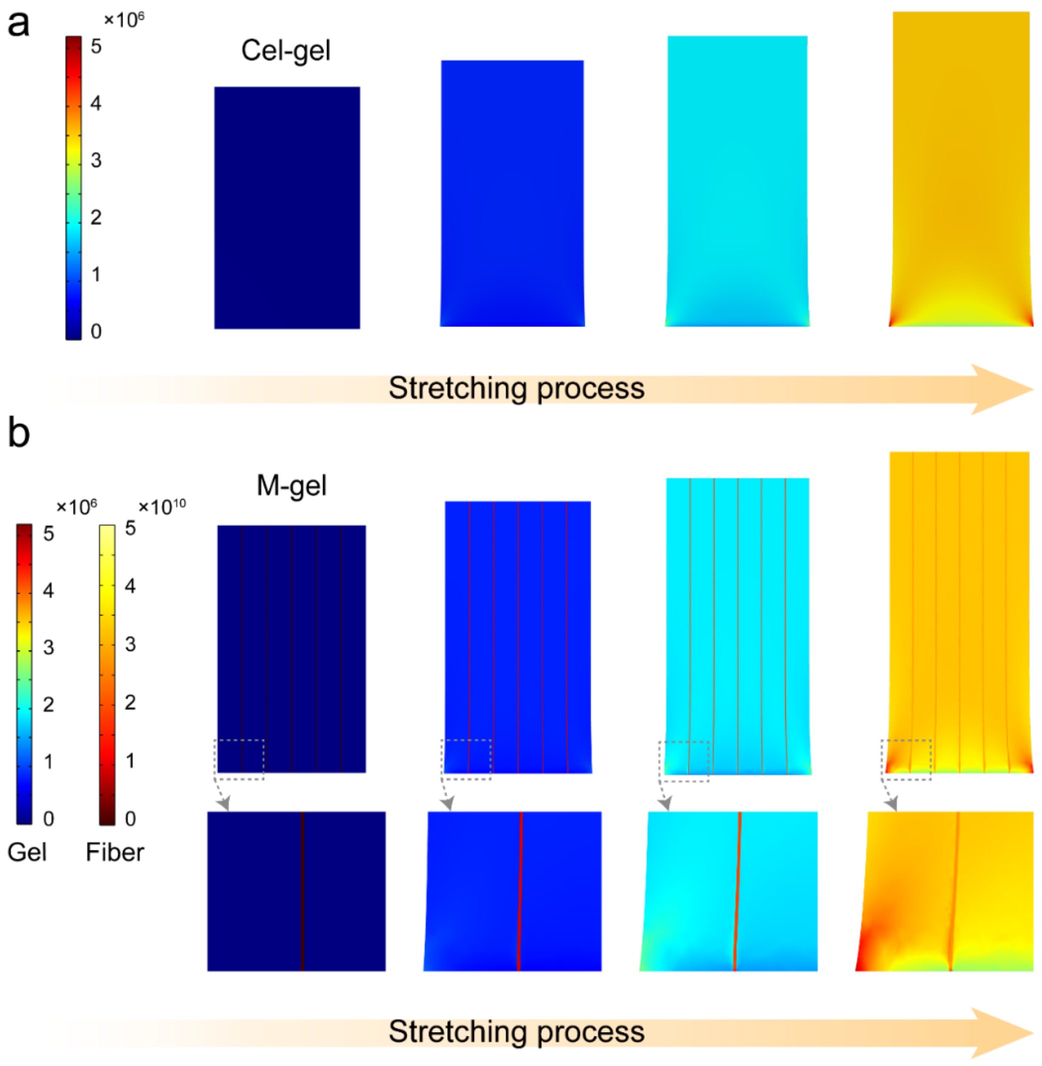

Figure S10. Tensile behavior diagrams of Cel-gel and M-gel simulated by the finite element. (a) Stress distribution nephogram of the Cel-gel during stretching process. (b) Stress distribution nephogram of the M-gel during stretching process.

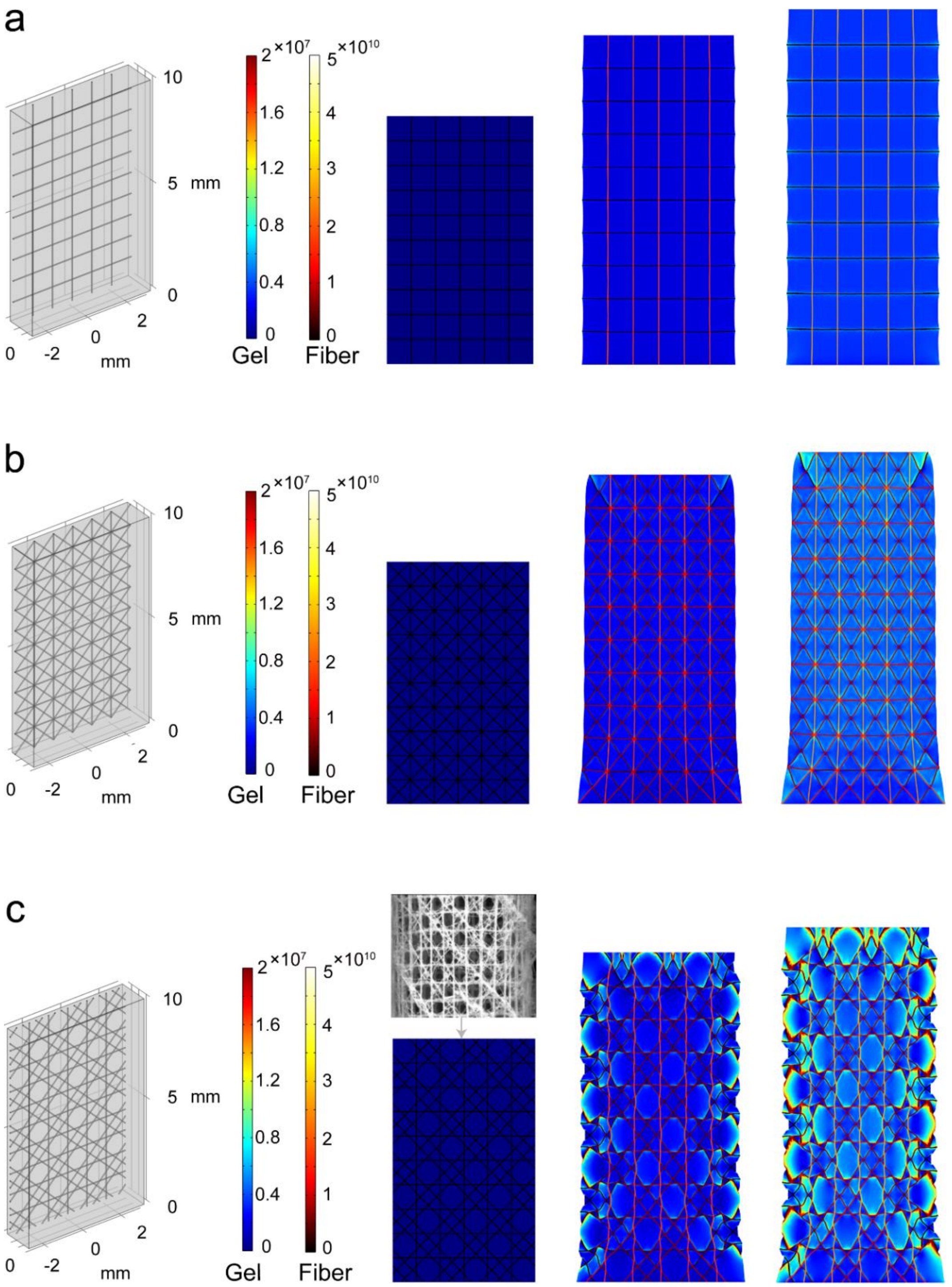

Figure S11. Tensile behavior diagrams of three kinds of M-gels by finite element simulations. (a) Modeling and stress distribution nephograms of the M-gel with cross grid during stretching process. (b) Modeling and stress distribution nephograms of the M-gel with diagonal grid during stretching process. (c) Modeling and stress distribution nephograms of the biomimetic M-gel during stretching process.

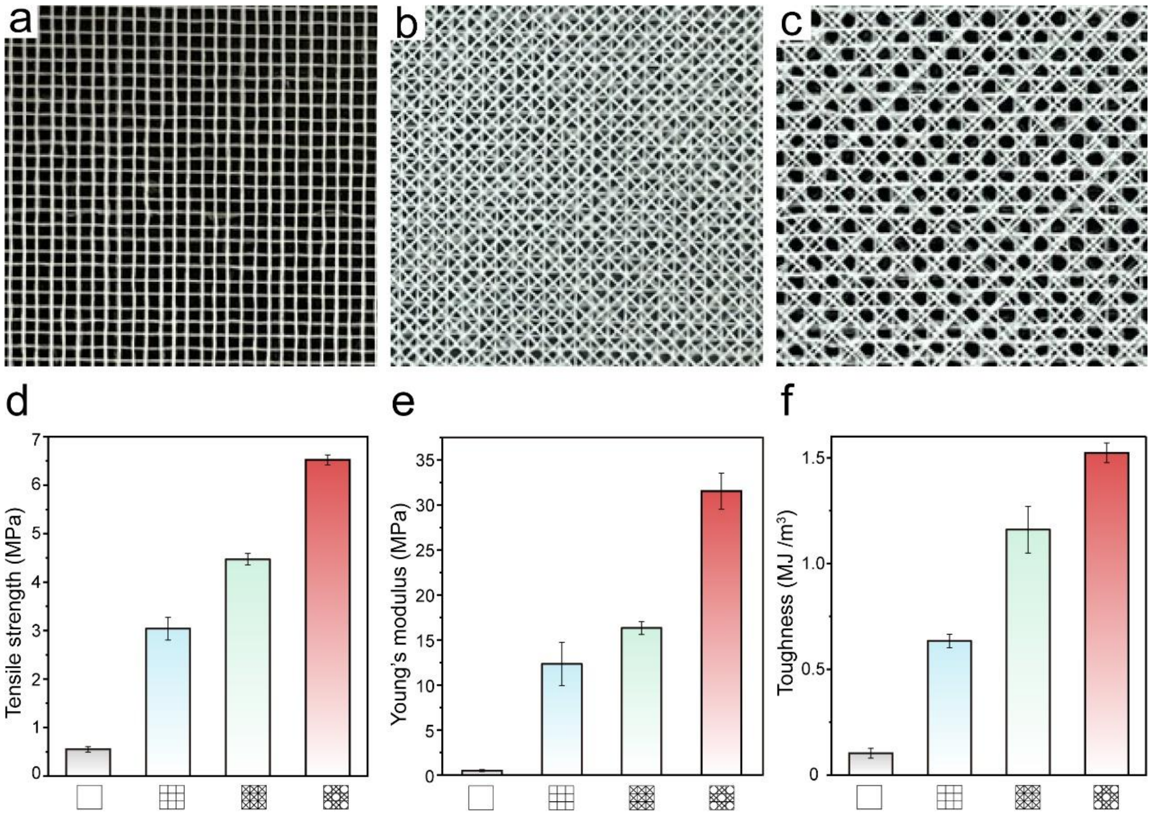

Figure S12. Optical images and mechanical properties of three kinds of M-gels. (a–c) Optical images of M-gel with cross grid, diagonal grid, and biomimetic grid architecture, respectively. Comparison of the (d) tensile strength, (e) Young's modulus, and (f) toughness of these three kinds of M-gels.

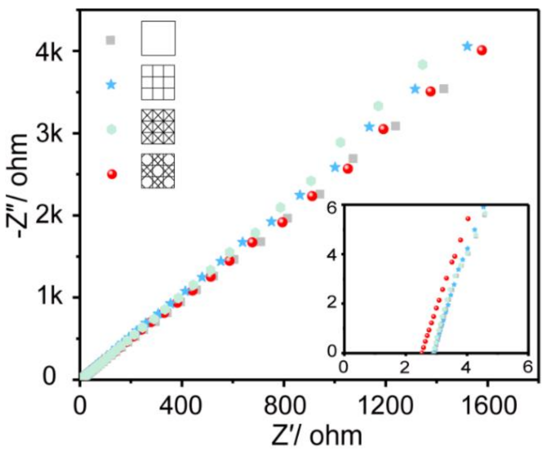

Figure S13. Electrochemical impedance spectroscopy curves (EIS) of Cel-gel and three kinds of M-gels.

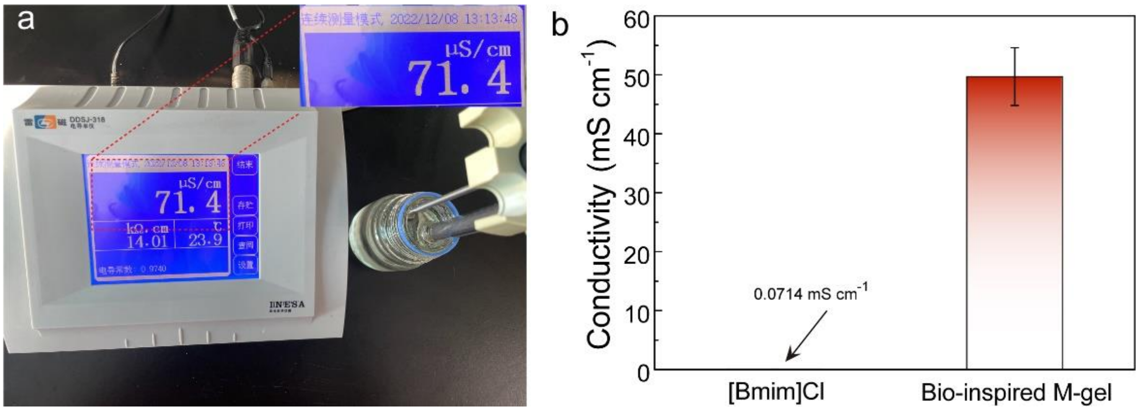

Figure S14. (a) Ionic conductivity of pure [Bmim]Cl ionic liquid. (b) Comparison of the ionic conductivity between bio-inspired M-gel and [Bmim]Cl.

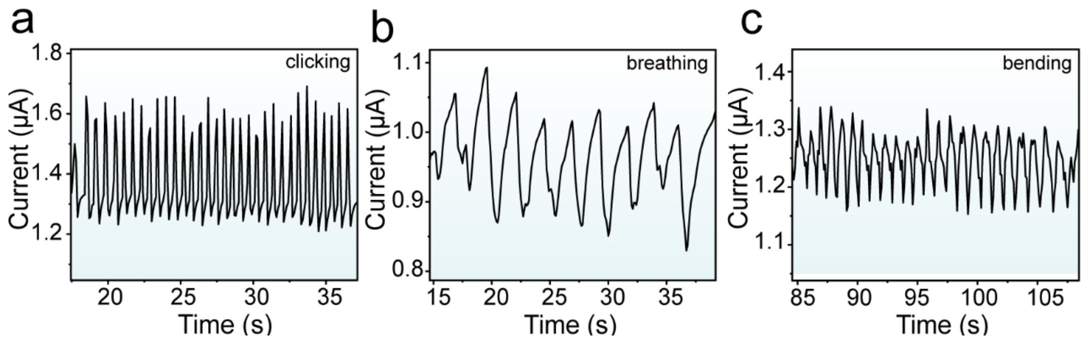

Figure S15. Potential applications of bio-inspired M-gels in robust, flexible bioelectronics. (a–c) Current waveforms of the bio-inspired M-gel sensing the clicking, breathing, and bending, respectively.

Table S1. Comparison of the bio-inspired multiscale design with other methods.

Gels	Preparation method	Mechanical property	Ionic conductivity	Potential application	Refs
Biomimetic M-gel	Bio-inspired multiscale design	Tensile strength of 6.5 MPa, elastic modulus of 31.5 MPa, impact resistance of 3.07 kJ/m	Conductivity of up to 49.6 mS/cm	Bioelectronic sensor and intelligent device	This work
Tough hydrogel	Regulating the assembly of silk nanofibers	Tensile stress of 1.55 MPa, elastic modulus of 5.88 MPa	/	Bioengineerin g	Ref. 1
Self-reinforced gel	Via the mechanical training strategy	Tensile strength of 4.7 MPa, elastic modulus of 21.3 MPa	/	Humidity-indu ced device	Ref. 2
Tunable Biomimetic Hydrogel	Photochemically crosslinked silk fibroin/cellulose	Tensile stress of 1.1 MPa, elastic modulus of ~14 MPa	/	3D printing inks and soft tissue	Ref. 3
Conductive regenerated gel	Via the double network design of silk fibroin and PAAm	Tensile strength of 1.17 MPa	Conductivity of 12 mS/cm	Strain sensor	Ref. 4
Strong and tough hydrogel	Freeze-casting–assisted solution substitution strategy	Tensile stress of 6.5 MPa, elastic modulus of 1.7 MPa	/	/	Ref. 5

Table S2. The information of the [Bmim]Cl ionic liquid, cellulose, and silk fibers for constructing the bio-inspired M-gel.

**Material**	**Amounts**	**Pretreatment**		**Aim**
[Bmim]Cl	94.1 wt%	80 °C	48 h	Removing water to dissolve cellulose
Cellulose	4.95 wt%	80 °C	48 h	Removing water for constructing the Cel-gel
Silk fiber	0.95 wt%	80 °C	48 h	Removing water for constructing the M-gel

References:

1. X. Zhang, L. Xiao, Z. Ding, Q. Lu, D. L. Kaplan. Engineered tough silk hydrogels through assembling β-Sheet rich nanofibers based on a solvent replacement strategy. *ACS Nano* **16**, 10209 (2022).
2. T. Shu, Z. Lv, C. T. Chen, G. X. Gu, J. Ren, L. Cao, Y. Pei, S. Ling, D. L. Kaplan. Mechanical training-driven structural remodeling: a rational route for outstanding highly hydrated silk materials. *Small* **17**, e2102660 (2021).
3. P. Dorishetty, R. Balu, S. S. Athukoralalage, T. L. Greaves, J. Mata, L. de Campo, N. Saha, A. C. W. Zannettino, N. K. Dutta, N. R. Choudhury. Tunable biomimetic hydrogels from silk fibroin and nanocellulose. *ACS Sustain. Chem. Eng*. **8**, 2375 (2020).
4. F. Chen, S. Lu, L. Zhu, Z. Tang, Q. Wang, G. Qin, J. Yang, G. Sun, Q. Zhang, Q. Chen. Conductive regenerated silk-fibroin-based hydrogels with integrated high mechanical performances. *J. Mater. Chem. B*. **7**, 1708 (2019).
5. X. Guo, X. Dong, G. Zou, H. Gao, W. Zhai. Strong and tough fibrous hydrogels reinforced by multiscale hierarchical structures with multimechanisms. *Sci. Adv*. **9**, eadf7075 (2023).
